# Self-Rated Health in Migrant and Non-Migrant Women before, during and after Pregnancy: A Population-Based Study of 0.5 Million Pregnancies from the Swedish Pregnancy Register

**DOI:** 10.3390/jcm9061764

**Published:** 2020-06-06

**Authors:** Pontus Henriksson, Emmie Söderström, Marie Blomberg, Paulina Nowicka, Kerstin Petersson, Kristin Thomas, Marcus Bendtsen, Fernando Estévez-López, Marie Löf

**Affiliations:** 1 Department of Health, Medicine and Caring Sciences, Linköping University, 581 83 Linköping, Sweden; emmie.soderstrom@liu.se (E.S.); kristin.thomas@liu.se (K.T.); marcus.bendtsen@liu.se (M.B.); marie.lof@liu.se (M.L.); 2 Department of Obstetrics and Gynaecology, and Department of Biomedical and Clinical Sciences, Linköping University, 581 83 Linköping, Sweden; marie.blomberg@liu.se; 3 Department of Food Studies, Nutrition and Dietetics, Uppsala University, 752 37 Uppsala, Sweden; paulina.nowicka@ikv.uu.se; 4 Department of Clinical Sciences, Obstetrics and Gynecology, Umeå University, 901 85 Umeå, Sweden; kerstin.petersson@skr.se; 5 Department of Child and Adolescent Psychiatry/Psychology, Erasmus MC University Medical Center, P.O. Box 2040, 3000 CA Rotterdam, The Netherlands; fer@estevez-lopez.com; 6 Department of Biosciences and Nutrition, Karolinska Institutet, 171 77 Stockholm, Sweden

**Keywords:** health inequalities, migration, pregnancy, self-rated health, self-reported health, self-perceived health

## Abstract

Self-rated health is a strong health marker. Migrants have been suggested to have poorer self-rated health than non-migrants (i.e., native-born). However, little is known about whether there are disparities in self-reported health in relation to pregnancy. Therefore, the aim of the current study was to examine the odds of poor self-rated health before, during and after pregnancy in migrant women as compared to women born in Sweden. We utilized population-based data from the Swedish Pregnancy Register containing 0.5 million women born in Sweden (i.e., non-migrant women) and migrant women between 2010 and 2018. Self-rated health was reported on a 5-point scale (from very poor to very good). Very poor and poor health were categorized as poor self-rated health. Logistic regression was utilized to calculate odds ratios (ORs) that were unadjusted and adjusted for covariates (age, parity, educational attainment and body mass index). The results demonstrate disparities in self-rated health across birth regions. In comparison to women born in Sweden, women born in Latin America and the Caribbean, South Asia as well as North Africa and the Middle East had consistently higher odds of poor self-rated health before, during and after pregnancy (ORs ranging from 1.14 to 1.96 in both unadjusted and adjusted models). Although women born in Sub-Saharan Africa did have comparable self-rated health as to women born in Sweden before pregnancy, after accounting for covariates, they had lower odds of poor self-rated health during and after pregnancy (ORs: 0.71 and 0.80 respectively). Therefore, additional measures and support may be needed to tackle disparities in health between migrant and non-migrant women before, during and after pregnancy.

## 1. Introduction

Self-rated health refers to the overall perception an individual has about their health and it is often measured by a one-item question, e.g., ‘How would you rate your general state of health?’ [1]. Albeit subjective, self-rated health by a one-item question has shown to be a useful marker of objectively measured health status [1,2,3,4,5,6,7]. Indeed, poor self-rated health has been associated with higher risk of mortality [1,2,3,4], cardiovascular disease [3,5], mental illness [4,6] and pregnancy complications [7]. For instance, a meta-analysis showed that individuals with poor self-rated health had twice the mortality risk as compared to persons with excellent self-rated health irrespectively of sex and follow-up time [1]. Self-rated health is typically used as a trustworthy indicator of public health and has been used to examine health disparities in various populations, e.g., [8,9,10,11]. 

Migrant health including reproductive health is a public health priority and an increasing proportion of the European adult population are first-generation migrants [12,13]. Pregnancy and the postpartum period are sensitive periods in time in which women may experience poor self-rated health [14], mental illness [15] as well as pregnancy complications [16,17]. Research has shown that migrant populations may have poorer self-rated health than the majority population (i.e., non-migrants) in European countries [11]. However, only a few studies have examined self-rated health in migrant and non-migrant women in relation to pregnancy [8,10]. To the best of our knowledge, no previous study has examined differences in self-reported health among migrant and non-migrant women at multiple time points before, during and after pregnancy in a large nationwide sample. The aim of this study was therefore to examine the odds of poor self-rated health before, during and after pregnancy in migrant women as compared to women born in Sweden. We utilized population-based data from the Swedish Pregnancy Register containing 0.5 million women born in Sweden (i.e., non-migrant women) and migrant women born in all seven so called super-regions [18]: (1) Central Europe, Eastern Europe and Central Asia; (2) high income countries (Sweden not included); (3) Latin America and the Caribbean; (4) North Africa and the Middle East; (5) South Asia; (6) Southeast Asia and East Asia; and (7) Sub-Saharan Africa. 

## 2. Methods

### 2.1. Study Design

We used population-based data from the Swedish Pregnancy Register with a coverage of approximately 90% of all births in Sweden [19]. Data from between 2010 and 2018 was utilized and encompassed 841,503 singleton pregnancies with mothers between 15 and 55 years of age. Of these pregnancies, there was missing data regarding maternal country of birth for 86,590 pregnancies and 125,005 pregnancies had missing data for covariates (i.e., educational attainment, parity and body mass index, BMI). Finally, there was missing data regarding self-rated health before (*n* = 52,143), during (*n* = 196,660) and after pregnancy (*n* = 203,484). Thus, the final analytic sample consisted of 577,765, 433,248 and 426,424 pregnancies before, during and after pregnancy, respectively. This study received ethical approval by the Regional Ethical Review Board, Stockholm, Sweden (2018/656-31). Since this is a registry study, data cannot be shared publicly due to legal reasons. Data could be made available to researchers after ethical approval (see https://etikprovningsmyndigheten.se and https://medscinet.com/gr/engelska.aspx).

### 2.2. Study Variables

Data regarding the women’s own birth country, age, parity, occupation and educational attainment (no education/elementary school, high school or university degree) was reported by the women at their first visit in antenatal care which is usually scheduled in the first trimester (around gestational week 8). Body height and weight were measured during this visit and BMI (kg/m^2^) was calculated as the body weight (kg) divided by height squared (m^2^). Data on the birth country of the women was categorized as follows: (1) Sweden; (2) Central Europe, Eastern Europe and Central Asia; (3) high income countries (Sweden not included); (4) Latin America and the Caribbean; (5) North Africa and the Middle East; (6) South Asia; (7) Southeast Asia and East Asia; and (8) Sub-Saharan Africa. Super-regions and other regions were categorized as described in the Global Burden of Disease study [18], which considers geographic closeness and epidemiological similarity for the creation of regions. Self-rated health before pregnancy was reported during the first visit in antenatal care whereas self-rated health during and after pregnancy was reported by the women at postpartum visit in antenatal care, 4 to 16 weeks after delivery. Women reported their self-rated health using a 5-point scale (i.e., very poor, poor, neither good or poor, good and very good). Very poor and poor health were categorized as poor self-rated health in the analysis. 

### 2.3. Statistical Analysis

We utilized logistic regression to calculate odds ratios (ORs), with corresponding 95% confidence intervals (CIs), for poor self-rated health before, during and after pregnancy by birth regions. Two logistic regression models were fitted: an unadjusted model and a model adjusting for maternal age, parity, educational attainment and BMI. Women born in Sweden were the reference group in all analyses. The statistical analysis was conducted using SPSS Statistics 22 (IBM, Arnmonk, NY, USA) and R version 3.6.1.

## 3. Results

### 3.1. Descriptive Data

Table 1 presents descriptive data of the women included in this study. The data showed variation in self-rated health across birth-regions. There was also a clear trend that self-rated health decreased during pregnancy and returned to similar levels as before pregnancy, postpartum. Data for 102 individual countries with ≥100 pregnancies is presented in Table S1. We also analyzed the proportion of women with data on self-rated health according to birth region (Table S2). Women born in Sweden had generally higher proportions of available data regarding self-rated health before (88.0%), during (69.8%) and after pregnancy (68.3%) whereas women born in Sub-Saharan Africa had generally lower proportions of available data before, during and after pregnancy (81.5%, 48.8% and 48.9%, respectively). 

### 3.2. Odds of Poor Self-Rated Health before Pregnancy

**Figure 1:**
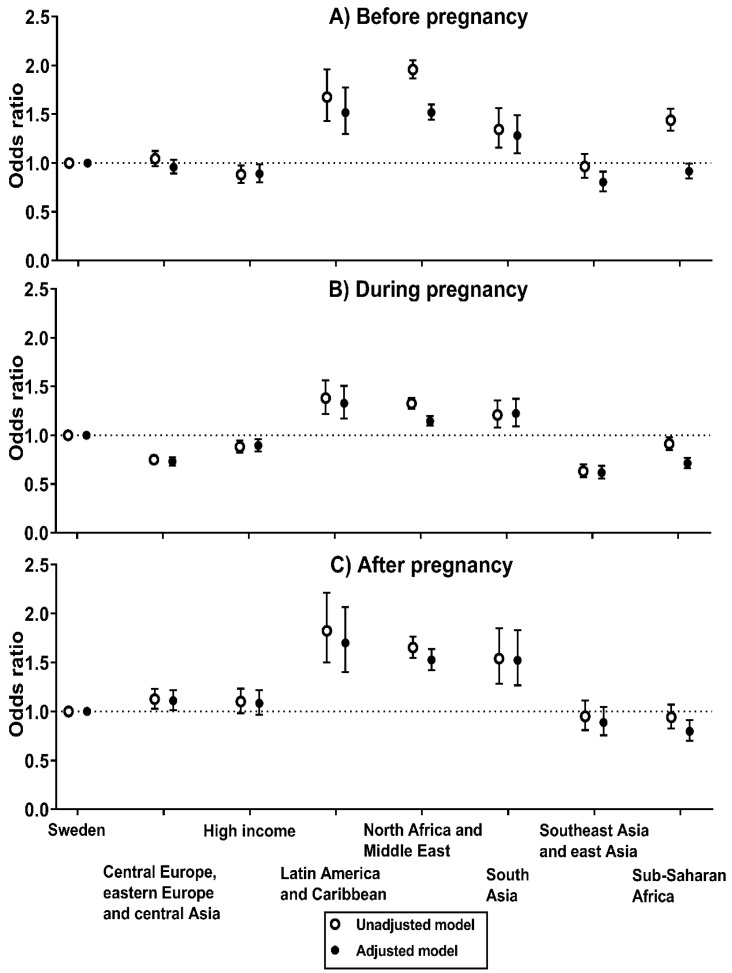
Odds of poor self-rated health (**A**) before pregnancy before pregnancy (*n* = 577,765), (**B**) during pregnancy (*n* = 433,248), and (**C**) after pregnancy (*n* = 426,424) by birth regions. Logistic regression was utilized to calculate odds ratios with 95% confidence intervals (women born in Sweden was the reference group). Adjusted models included maternal age, parity, educational attainment and body mass index as covariates.

Figure 1A shows the odds of poor self-rated health before pregnancy by birth regions (detailed data is presented in Table S3). In the unadjusted model, women born in Latin America and the Caribbean (OR, 1.68 [95% CI, 1.43 to 1.96]), North Africa and the Middle East (OR, 1.96 [95% CI, 1.87 to 2.05]) and South Asia (OR, 1.34 [95% CI, 1.16 to 1.56]) had considerably higher odds of having poor self-rated health before pregnancy as compared to women born in Sweden. Although the estimates generally were attenuated in the adjusted model, women born in Latin America and the Caribbean (adjusted OR, 1.52 [95% CI, 1.30 to 1.76]), North Africa and the Middle East (adjusted OR, 1.52 [95% CI, 1.44 to 1.60]) and South Asia (adjusted OR, 1.28 [95% CI, 1.10 to 1.49]) still had higher odds of poor self-rated health than women born in Sweden. Women born in Sub-Saharan Africa had higher odds of poor self-rated health than women born in Sweden in the unadjusted model (OR, 1.44 [95% CI, 1.33 to 1.56]). However, after adjustments for maternal age, parity, educational attainment and BMI, women born in Sub-Saharan Africa had slightly lower odds of poor self-rated health (adjusted OR, 0.92 [95% CI, 0.84 to 0.99]) as compared to women born in Sweden.

### 3.3. Odds of Poor Self-Rated Health during Pregnancy

During pregnancy, women born in Latin America and the Caribbean (adjusted OR, 1.33 [95% CI, 1.17 to 1.51]), South Asia (adjusted OR, 1.22 [95% CI, 1.09 to 1.37]) and North Africa and the Middle East (adjusted OR, 1.14 [95% CI, 1.10 to 1.20]) had higher odds of poor self-rated health than women born in Sweden, both in the unadjusted and adjusted model (Figure 1B) (detailed data in Table S3). Conversely, women born in Sub-Saharan Africa had lower odds of poor self-rated heath both in the unadjusted and adjusted model (adjusted OR, 0.71 [95% CI, 0.66 to 0.77]).

### 3.4. Odds of Poor Self-Rated Health after Pregnancy

The ORs of poor self-rated health after pregnancy by birth regions are presented in Figure 1C (detailed data in Table S3). Both in the unadjusted and adjusted model, women born in Latin America and the Caribbean (adjusted OR, 1.70 [95% CI, 1.40 to 2.07]), South Asia (adjusted OR, 1.52 [95% CI, 1.27 to 1.83]) and North Africa and the Middle East (adjusted OR, 1.53 [95% CI, 1.42 to 1.64]) had higher odds of poor self-rated health after pregnancy as compared to women that were born in Sweden. On the contrary, women born in Sub-Saharan Africa had lower odds of poor self-rated heath (adjusted OR, 0.80 [95% CI, 0.70 to 0.91]).

### 3.5. Sensitivity Analyses

We also performed a series of sensitivity analyses to assess the robustness of our findings. First, we examined whether the ORs of poor self-rated health before pregnancy would change if we only utilized data for pregnancies with existing data of self-rated health for all three time points (before, during and after pregnancy). As shown in Figure S1, the ORs were comparable to our main analysis of poor self-rated health before pregnancy. Second, as shown in Table S4, we also included an additional adjustment for maternal occupation in the adjusted model for poor self-rated health before, during and after pregnancy, and estimates remained virtually the same as in the adjusted models. Third, we estimated a generalized linear mixed model with a time by region interaction to corroborate our findings and to analyze changes in self-rated health by birth regions (Table S5). The results showed that the ORs for poor self-rated health before pregnancy were similar across birth regions as compared to our main analyses (i.e., Figure 1A). Regarding changes during and after pregnancy, women born in North Africa and the Middle East as well as Sub-Saharan Africa both had a decrease in the ORs for poor self-rated health which also agrees with the findings presented in Figure 1 (please see Table S5 for all results). Forth, we examined whether the prevalence of poor self-rated health changed during the study years. Our analyses showed that the prevalence of poor self-rated health before (3.0% vs. 2.8%), during (8.6% vs. 8.6%) and after pregnancy (2.7% vs. 2.4%) was comparable between 2010 and 2014 as compared to between 2015 and 2018, respectively. Furthermore, associations of birth regions with self-rated health were generally very comparable between 2010 and 2014 versus between 2015 and 2018. Fifth, we also adjusted associations for premature birth (delivery < 37 gestational weeks) but results remained virtually identical. Sixth, we performed an additional adjustment for the use of an interpreter during the visits in antenatal care (yes vs. no), which may serve as a surrogate measure for acculturation, but results and conclusions remained the same. Finally, we also adjusted our results for the county of the maternity care and results remained similar to our main results. 

## 4. Discussion

### 4.1. Main Findings

This population-based study of over 0.5 million pregnancies in Sweden demonstrates disparities in self-rated health across birth regions. In comparison to women born in Sweden, women born in Latin America and the Caribbean, South Asia as well as North Africa and the Middle East had consistently higher odds of poor self-rated health before, during and after pregnancy. Interestingly, although women born in Sub-Saharan Africa did have comparable self-rated health as to women born in Sweden before pregnancy, after adjusting for covariates, they had lower odds of poor self-rated health during and after. 

### 4.2. Interpretation

Only a few previous studies have examined self-rated health in migrant and non-migrant women in relation to pregnancy [8,10]. These studies have only utilized single time points which complicates comparison to our findings before, during and after pregnancy. Liu et al. reported that migrant women born in the five most prevalent countries of emigration (i.e., Syria, Iraq, Somalia, Eritrea or Afghanistan) had greater risk of poor self-reported health before pregnancy than women born in Sweden [10]. These findings are in line with our results although our results (including the country-specific results in Table S1) indicate that women born in Syria, Iraq and Afghanistan (i.e., the Middle East) have worse self-rated health than women born in Somalia and Eritrea (i.e., Sub-Saharan Africa). Furthermore, a French study by El-Khory et al. found that women born in North Africa and Turkey, but not Sub-Saharan Africa, had statistically significant higher odds of poor self-rated health two months postpartum as compared to women born in France or the EU [8,10]. These results can be reconciled with our findings showing that women born in North Africa and the Middle East have considerably greater odds of poor self-rated health postpartum compared to women born in Sweden, whereas women born in Sub-Saharan Africa had comparable or slightly lower odds. Furthermore, we found no previous studies that reported data for self-reported health of women born in South Asia or Latin America which is of importance considering that women born in these regions consistently had greater odds of poor self-reported health before, during and after pregnancy. Finally, we also extend the literature by examining differences in self-rated health before, during and after pregnancy for all super-regions and 102 countries of birth. 

Our results may also be compared to European surveys of women in working age. Such studies have found that migrant women born in North Africa and the Middle East [20] including specific countries such as Iraq [21,22], Iran [22,23], Turkey [22,23] and Lebanon [22] to have worse self-reported health than non-migrants. There is less data regarding women born in other regions than North Africa and the Middle East even though a previous Swedish study reported that women born in Latin America, but not Sub-Saharan Africa had greater odds of poor self-rated health than women born in Sweden [20] which confirms our findings. Although our observed inequalities in self-reported health were generally attenuated after adjustments for covariates including educational attainment, they remained higher than for women born in Sweden. This observation is in line with previous studies conducted in non-pregnant women suggesting that differences in self-reported health between migrants and non-migrants may be attenuated but not diminished after adjusting for socioeconomic status indicators [20,22]. 

We also found birth regions that had associations with better self-reported health. For instance, women born in Southeast Asia and East Asia had consistently slightly lower odds of poor self-reported health before, during and after pregnancy. Interestingly, women born in Sub-Saharan Africa had higher or comparable odds of poor self-rated health before pregnancy compared to women born in Sweden, but comparable or lower odds during and after pregnancy. A potential reason why women born in Sub-Saharan Africa may have worse self-rated health before pregnancy but better self-rated health during and after pregnancy is due to attrition, i.e., that women born in Sub-Saharan Africa with poor self-reported health are lost during follow-up. However, the results were identical in our sensitivity analyses which only included women with complete data including the follow-ups. Another reason for the interesting results could be that pregnancy and childbirth may be a protective factor given the cultural importance of childbirth and family with the accompanying social support given to the pregnant women, e.g., [24,25,26] although further studies are needed. 

### 4.3. Strengths and Limitations

The main strength of this study was the population-based sample of women from the Swedish Pregnancy Register which covers approximately 90% of all birth in Sweden [19]. Furthermore, the large study sample of over 0.5 million women in a multiethnic country allowed analysis of all super-regions with large samples sizes for all regions (all *n* > 3000).

This study also has some limitations that need to be acknowledged. First, self-rated health before, during and after pregnancy was examined using a one-item question. However, previous studies of self-rated health have showed strong associations between self-rated health and health outcomes in different populations and ethnic groups [1,27,28] using a one-item question. Second, as for all studies examining potentially vulnerable groups there is a risk of attrition, i.e., that migrant women with poor self-rated health are not included in the data which may underestimate the prevalence of poor self-reported health. Our analyses (Table S2) showed that the registry contained generally more complete data on self-reported health in women that were born in Sweden as compared to other birth regions such as Sub-Saharan Africa. Our sample was, however, population-based and the sensitivity analyses showed that the results for the self-reported health before pregnancy were similar even when only those who had data at all three time points were included. However, further studies should address the role of attrition for the results. Third, we only had data on first-generation migrants and did not have any data regarding the reason for migration. Thus, future studies could consider self-rated health among first and second-generation migrants, reasons for migration and how these factors potentially relate to self-reported health. Forth, although we adjusted for premature birth in a sensitivity analyses, future studies should consider other adverse pregnancy outcomes in relation to self-rated health. Finally, we did not have any data regarding acculturation or length of residency although results remained virtually the same after adjusting for the use of a professional interpreter in maternity care (as a crude proxy for acculturation). 

### 4.4. Clinical and Public Health Implications

Our results demonstrate distinct disparities in self-rated health. In particular, women born in Latin America and the Caribbean, South Asia as well as North Africa and the Middle East had consistently poorer self-reported health before, during and after pregnancy. These disparities may be of both clinical and public health importance considering that poor self-reported health is a strong risk factor for later mortality and morbidity [1,2,3,4,5,6,7]. Thus, the findings indicate that migrant women experience poor self-rated health before, during and after pregnancy which motivate additional efforts to promote health but may also provide an opportunity to intervene considering the regular appointments in antenatal care. Previous literature has identified some probable causes of poor self-rated health in migrant non-pregnant populations such as discrimination, low social support and unhealthy health behaviors, e.g., [29,30,31]. However, further studies of the determinants of self-rated health before, during and after pregnancy may be needed in order to provide optimal support and care during pregnancy as well as to form a basis for future health promotion. Furthermore, additional studies are warranted considering that associations of birth regions and self-rated health in migrants may differ due to the country of residence and its integration policies [32,33]. 

## 5. Conclusions

This population-based study of over 0.5 million pregnancies from the Swedish Pregnancy Register demonstrates disparities in self-rated health across birth regions. For instance, women born in Latin America and the Caribbean, South Asia as well as North Africa and the Middle East had consistently higher odds of poor self-rated health before, during and after pregnancy as compared to women born in Sweden. Our results indicate that additional measures and support may be needed to tackle disparities in health between migrant and non-migrant women before, during and after pregnancy.

## Figures and Tables

**Table 1:** Descriptive data of the women in this study.

Characteristics	Sweden	Central Europe, Eastern Europe and Central Asia ^1^	High Income Countries ^2^	Latin America and the Caribbean ^3^	North Africa and the Middle East ^4^	South Asia ^5^	Southeast Asia and East Asia ^6^	Sub-Saharan Africa ^7^
n (%)	456,045	28,180	16,308	3732	40,172	4948	9877	18,503
	(78.9%)	(4.9%)	(2.8%)	(0.6%)	(7.0%)	(0.9%)	(1.7%)	(3.2%)
Age (years)	30.7 ± 5.0	30.6 ± 5.2	32.7 ± 5.0	32.2 ± 5.3	30.1 ± 4.5	30.5 ± 4.5	32.1 ± 5.1	30.3 ± 5.7
Primiparous	45.1%	43.3%	44.6%	43.6%	35.1%	47.5%	44.8%	29.5%
Educational attainment								
No or elementary school	4.4%	13.3%	4.6%	9.4%	29.1%	12.4%	24.4%	50.9%
High school	41.1%	41.5%	25.5%	41.7%	37.9%	28.9%	36.2%	35.2%
University	54.5%	45.2%	69.9%	48.8%	33.0%	58.7%	39.4%	13.8%
BMI (kg/m^2^)	24.8 ± 4.7	24.2 ± 4.4	24.5 ± 4.7	25.2 ± 4.4	25.7 ± 4.6	24.8 ± 4.4	22.8 ± 3.8	26.1 ± 5.3
Poor self-reported health								
Before pregnancy	2.7%	2.8%	2.4%	4.4%	5.1%	3.6%	2.6%	3.8%
During pregnancy ^8^	8.6%	6.6%	7.7%	11.5%	11.1%	10.2%	5.6%	7.9%
After pregnancy ^9^	2.5%	2.8%	2.7%	4.4%	4.0%	3.8%	2.3%	2.3%

Data is presented as the mean ± standard deviation or as %. BMI, body mass index. ^1^ Included regions/countries were: Central Asia (Armenia, Azerbaijan, Georgia, Kazakhstan, Kyrgyzstan, Mongolia, Tajikistan, Turkmenistan and Uzbekistan); Central Europe (Albania, Bosnia and Herzegovina, Bulgaria, Croatia, Czech Republic, Hungary, Macedonia, Montenegro, Poland, Romania, Serbia, Slovakia and Slovenia); Eastern Europe (Belarus, Estonia, Latvia, Lithuania, Moldova, Russia and Ukraine). ^2^ Included countries were: Australasia (Australia and New Zealand); high-income Asia Pacific (Brunei, Japan, Singapore and South Korea); high-income North America (Canada, Greenland and USA); Southern Latin America (Argentina, Chile and Uruguay); Western Europe (Andorra, Austria, Belgium, Cyprus, Denmark, Finland, France, Germany, Greece, Iceland, Ireland, Israel, Italy, Luxembourg, Malta, Netherlands, Norway, Portugal, Spain, Switzerland and UK). ^3^ Included regions/countries were: Andean Latin America (Bolivia, Ecuador, Peru); the Caribbean (Antigua and Barbuda, The Bahamas, Barbados, Belize, Bermuda, Cuba, Dominica, Dominican Republic, Grenada, Guyana, Haiti, Jamaica, Puerto Rico, Saint Lucia, Saint Vincent and the Grenadines, Suriname, Trinidad and Tobago and Virgin Islands); Central Latin America (Colombia, Costa Rica, El Salvador, Guatemala, Honduras, Mexico, Nicaragua, Panama and Venezuela); Tropical Latin America (Brazil and Paraguay). ^4^ Included regions/countries were: Afghanistan, Algeria, Bahrain, Egypt, Iran, Iraq, Jordan, Kuwait, Lebanon, Libya, Morocco, Oman, Palestine, Qatar, Saudi Arabia, Sudan, Syria, Tunisia, Turkey, United Arab Emirates and Yemen. ^5^ Included regions/countries were: Bangladesh, Bhutan, India, Nepal and Pakistan. ^6^ Included regions/countries were: East Asia (China, North Korea and Taiwan (province of China)); Southeast Asia (Cambodia, Indonesia, Laos, Malaysia, Maldives, Mauritius, Myanmar, Philippines, Sri Lanka, Seychelles, Thailand, Timor-Leste and Vietnam). Oceania was not included due too few observations. ^7^ Included regions/countries were: Central Sub-Saharan Africa (Angola, Central African Republic, Democratic Republic of the Congo, Equatorial Guinea and Gabon); Eastern Sub-Saharan Africa (Burundi, Comoros, Djibouti, Eritrea, Ethiopia, Kenya, Madagascar, Malawi, Mozambique, Rwanda, Somalia, South Sudan, Tanzania, Uganda and Zambia); Southern Sub-Saharan Africa (Botswana, Lesotho, Namibia, South Africa, Swaziland (eSwatini) and Zimbabwe); Western Sub-Saharan Africa (Benin, Burkina Faso, Cameroon, Cape Verde, Chad, Côte d’Ivoire, The Gambia, Ghana, Guinea, Guinea-Bissau, Liberia, Mali, Mauritania, Niger, Nigeria, São Tomé and Príncipe, Senegal, Sierra Leone and Togo). ^8^ Number of pregnancies with data on self-reported health during pregnancy were; Sweden (*n* = 354,242); Central Europe, Eastern Europe and Central Asia (*n* = 18,859); high income countries (*n* = 11,652), Latin America and the Caribbean (*n* = 2456), North Africa and the Middle East (*n* = 25,257), South Asia (*n* = 3206), Southeast Asia and East Asia (*n* = 6830), Sub-Saharan Africa (*n* = 10,746). ^9^ Number of pregnancies with data self-reported health after pregnancy were; Sweden (*n* = 347,562), Central Europe, Eastern Europe and Central Asia (*n* = 18,783), high income countries (*n* = 11,587), Latin America and the Caribbean (*n* = 2448), North Africa and the Middle East (*n* = 25,253), South Asia (*n* = 3200), Southeast Asia and East Asia (*n* = 6812), Sub-Saharan Africa (*n* = 10,779).

## Supplementary Materials

The following are available online at https://www.mdpi.com/2077-0383/9/6/1764/s1, Table S1: Poor self-rated health before, during and after pregnancy according to birth country (countries/regions with ≥100 pregnancies). Table S2: Proportion of women with data of self-rated health according to birth regions. Table S3: Odds ratios with 95% confidences intervals for poor self-rated health before, during and after pregnancy by birth regions. Figure S1: Sensitivity analysis showing the odds ratios of poor self-rated health before pregnancy by birth regions for women with complete data of self-rated health at all time points, i.e., before, during and after pregnancy (*n* = 398,025). Table S4: Odds ratios with 95% confidences intervals for poor self-rated health before (*n* = 576,491), during (*n* = 432,266) and after pregnancy (*n* = 425,468) by birth regions with adjustments for maternal age, parity, educational attainment, body mass index as well as occupation. Table S5: Generalized linear mixed model with a time by region interaction for self-rated health before, during and after pregnancy.
