# Supplementary material for: Self-Rated Health in Migrant and Non-Migrant Women before, during and after Pregnancy: A Population-Based Study of 0.5 Million Pregnancies from the Swedish Pregnancy Register
Source: J Clin Med. 2020 Jun 6;9(6):1764. doi: 10.3390/jcm9061764 (PMC7356601; doi:10.3390/jcm9061764)
Supplement: Supplementary file 1 [file jcm-09-01764-s001.pdf]

Supplementary Material

Self-rated health in migrant and non-migrant women before, during and after pregnancy

- a population-based study of 0.5 million pregnancies from the Swedish Pregnancy Register

Pontus Henriksson, Emmie Söderström, Marie Blomberg, Paulina Nowicka, Kerstin Petersson, Kristin Thomas, Marcus Bendtsen, Fernando Estévez-López, Marie Löf

Corresponding author: Pontus Henriksson, Department of Health, Medicine and Caring Sciences, Linköping University, Linköping 58183, Sweden.

Phone: 46-13281000; Email: pontus.henriksson@liu.se.

Table S1. Poor self-rated health before, during and after pregnancy according to birth country (countries/regions with ≥ 100 pregnancies).

	Poor self-rated health					
**Birth region/county**	**n**	**Before pregnancy (%)**	**n**	**During pregnancy (%)**	**n**	**After pregnancy (%)**
**All countries**	577 765	2.9	433 248	8.6	426 424	2.6
**Sweden**	456 045	2.7	354 242	8.6	347 562	2.5
**Central Europe, Eastern Europe and Central Asia**	28 180	2.8	18 859	6.6	18 783	2.8
Central Asia	2 207	5.2	1 187	6.3	1 196	3.0
Armenia	350	5.1	214	11.2	219	5.0
Azerbaijan	272	6.6	167	8.4	170	2.9
Georgia	145	3.4	69	8.7	69	2.9
Kazakhstan	244	8.2	133	8.3	133	5.3
Kyrgyzstan	141	4.3	78	2.6	78	2.6
Mongolia	523	4.4	259	1.2	260	1.9
Uzbekistan	466	4.3	230	5.7	229	1.3
Central Europe	20 124	2.7	13 795	7.2	13 723	2.9
Albania	1 665	4.1	1 138	9.6	1 127	3.7
Bosnia and Herzegovina	4 873	2.7	3 518	8.1	3 488	3.8
Bulgaria	601	2.0	413	6.1	411	1.2
Croatia	831	2.9	588	9.0	578	1.7
Czech Republic	226	0.9	169	3.0	162	1.9
Hungary	649	3.5	477	5.5	480	1.9
Macedonia	908	2.2	642	7.3	648	2.5
Montenegro	386	3.9	277	6.5	278	4.0
Poland	5 330	2.2	3 503	5.9	3 476	2.2
Romania	1 842	2.6	1 259	6.7	1 262	2.9
Serbia	2 501	2.9	1 585	8.1	1 590	3.4
Slovakia	189	3.2	131	1.5	129	3.1
Slovenia	123	1.6	95	6.3	94	1.1
Eastern Europe	5 849	2.3	3 877	4.4	3 864	2.1
Belarus	306	1.3	200	2.0	198	3.0
Estonia	606	1.5	412	5.1	414	1.2
Latvia	620	1.5	418	5.7	415	2.7
Lithuania	1 089	1.5	770	3.1	768	1.2
Moldova	128	0	85	5.9	84	3.6
Russia	2 167	3.1	1 379	5.2	1 377	2.8
Ukraine	933	3.0	613	3.4	608	1.5
**High income countries** (Sweden not included)	16 308	2.4	11 652	7.7	11 587	2.7
Australasia	243	1.6	158	7.0	154	3.9
Australia	200	1.5	127	7.9	124	4.0
High-income Asia Pacific	1 137	2.0	833	6.4	824	3.4
Japan	342	0.3	240	4.6	237	2.5
South Korea	739	2.6	550	7.3	545	3.7
High-income North America	999	1.6	679	8.4	676	3.8
Canada	225	0.4	138	11.6	141	4.3
USA	766	1.8	535	7.7	529	3.8
Southern Latin America	1 795	4.1	1 164	11.2	1 168	3.5
Argentina	194	4.1	133	9.0	134	0.7
Chile	1 524	4.2	977	11.3	980	3.8

Table S1, continued…

	Poor self-rated health					
**Birth region/county**	**n**	**before pregnancy (%)**	**n**	**during pregnancy (%)**	**n**	**after pregnancy (%)**
Western Europe	12 134	2.2	8 818	7.3	8 765	2.4
Austria	189	3.2	141	5.7	138	2.2
Belgium	133	3.8	104	7.7	101	1.0
Denmark	1 369	2.9	1 035	7.6	1 036	2.5
Finland	2 263	2.0	1 620	7.5	1 609	2.2
France	549	1.6	411	6.8	404	2.2
Germany	2 284	1.8	1 754	6.6	1 748	2.5
Greece	691	1.4	474	6.8	472	2.8
Iceland	474	3.0	340	10.6	341	1.5
Ireland	114	2.6	73	5.5	72	0
Israel	163	3.7	106	11.3	104	3.8
Italy	417	2.2	296	4.1	291	2.4
Netherlands	382	3.1	297	6.4	289	2.4
Norway	1 290	1.9	933	9.1	925	2.9
Portugal	143	2.8	92	3.3	93	3.2
Spain	620	2.7	413	8.0	414	2.7
Switzerland	156	3.2	109	2.8	108	0.9
UK	695	2.4	483	8.1	481	2.9
**Latin America and Caribbean**	3 732	4.4	2 456	11.5	2 448	4.4
Andean Latin America	992	6.3	600	12.5	596	4.4
Bolivia	285	8.8	154	13.0	148	5.4
Ecuador	156	4.5	98	11.2	95	1.1
Peru	551	5.4	348	12.6	353	4.8
Caribbean	291	3.8	170	12.9	170	4.1
Cuba	157	2.5	82	12.2	81	7.4
Central Latin America	1 725	3.9	1 183	10.9	1 186	4.0
Colombia	899	4.1	617	11.0	617	3.6
El Salvador	242	5.8	161	9.9	161	5.0
Mexico	221	1.8	162	8.0	164	4.3
Venezuela	129	6.2	88	17.0	89	5.6
Tropical Latin America	724	3.3	503	11.3	496	5.6
Brazil	701	3.3	486	10.9	481	5.6
**North Africa and Middle East**	40 172	5.1	25 257	11.1	25 253	4.0
Afghanistan	2 662	7.0	1 696	12.4	1 704	6.6
Algeria	342	3.8	196	8.2	193	4.1
Egypt	570	5.6	346	9.5	352	5.4
Iran	4 059	5.2	2 497	13.4	2 494	4.5
Iraq	13 254	4.7	8 469	11.5	8 440	3.8
Jordan	608	2.8	356	11.2	360	3.9
Kuwait	145	3.4	98	10.2	101	4.0
Lebanon	2 555	5.8	1 768	13.4	1 746	4.6
Libya	241	2.9	134	13.4	134	6.0
Morocco	1 423	5.3	880	10.9	871	4.5
Palestine	858	5.7	540	10.6	540	3.5
Saudi Arabia	303	4.3	169	11.8	174	2.9
Sudan	434	3.7	259	7.3	261	1.1
Syria	7 714	5.7	4 977	8.4	5 010	2.9
Tunisia	558	5.0	302	11.6	301	4.3
Turkey	4 134	4.3	2 361	11.5	2 359	4.3

Table S1, continued…

	Poor self-rated health					
**Birth region/county**	**n**	**before pregnancy (%)**	**N**	**during pregnancy (%)**	**n**	**after pregnancy (%)**
United Arab Emirates	107	5.6	76	7.9	77	2.6
Yemen	189	5.8	121	6.6	125	4.0
**South Asia**	4 948	3.6	3 206	10.2	3 200	3.8
Bangladesh	797	6.5	473	14.4	475	4.8
India	2 257	2.7	1 576	10.7	1 568	3.9
Pakistan	1 809	3.4	1 101	8.2	1 102	3.2
**Southeast Asia and East Asia**	9877	2.6	6 830	5.6	6 812	2.3
East Asia	2574	1.9	1 822	2.4	1 831	2.3
China	2 480	1.9	1 750	2.3	1 759	2.3
Southeast Asia	7303	2.8	5 008	6.8	4 981	2.3
Indonesia	287	2.8	217	9.7	219	3.2
Philippines	1 420	3.0	969	5.1	963	2.6
Sri Lanka	891	3.1	603	13.1	600	3.3
Thailand	3 196	2.5	2 129	6.0	2 121	1.7
Vietnam	1 251	3.0	892	5.7	881	2.6
**Sub-Saharan Africa**	18 503	3.8	10 746	7.9	10 779	2.3
Central sub-Saharan Africa	553	5.8	340	16.2	337	3.3
Democratic Republic of the Congo	479	6.1	290	17.2	287	3.8
Eastern Sub-Saharan Africa	15 590	3.7	8 881	7.3	8 920	2.3
Burundi	411	3.9	290	9.7	290	2.1
Eritrea	3 318	3.9	1 899	7.3	1 904	2.1
Ethiopia	1 462	4.0	866	9.5	867	2.7
Kenya	393	3.8	248	5.6	252	2.4
Somalia	9 166	3.6	5 060	6.9	5 083	2.3
Tanzania	195	3.6	120	8.3	121	2.5
Uganda	410	2.9	248	7.3	250	4.0
Southern Sub-Saharan Africa	155	0	104	7.7	100	1.0
South Africa	105	0	75	8.0	72	1.4
Western Sub-Saharan Africa	2 205	4.7	1 421	9.9	1 422	2.4
Cameroon	222	5.4	145	13.8	145	0.7
The Gambia	493	4.3	304	9.2	298	1.7
Ghana	292	4.1	203	7.4	204	2.9
Nigeria	641	3.9	426	8.7	426	2.6
Sierra Leone	113	4.4	66	9.1	69	1.4

Table S2. Proportion of women with data of self-rated health according to birth regions.

	n[1]	Data of self-rated health		
		**Before pregnancy**	During pregnancy	**After** pregnancy
Sweden	577 919	88.0 %	69.8 %	68.3 %
Central Europe, Eastern Europe and Central Asia	38 450	87.7 %	60.0 %	59.6 %
High income countries	20 952	88.7 %	64.6 %	64.2 %
Latin America and Caribbean	5 150	87.0 %	58.4 %	58.2 %
North Africa and Middle East	59 818	84.5 %	55.6 %	55.6 %
South Asia	6 898	85.4 %	57.1 %	56.9 %
Southeast Asia and East Asia	14 503	85.7 %	61.2 %	60.9 %
Sub-Saharan Africa	31 223	81.5 %	48.8 %	48.9 %
No reported birth country[2]	86 590	29.0 %	26.9 %	17.7 %

[1] Please note that the n is higher than the final analytic samples since pregnancies without data regarding birth country and covariates were excluded in the final analytic sample.

[2] The prevlance of poor self-rated health for those without a reported birth county was 4.4 % before, 10.1 % during and 3.0 % after pregnancy.

Table S3. Odds ratios with 95 % confidences intervals for poor self-rated health before, during and after pregnancy by birth regions.

	Poor self-rated health					
	**Before pregnancy**		**During pregnancy**		**After pregnancy**	
	Unadjusted	Adjusted[1]	Unadjusted	Adjusted[1]	Unadjusted	Adjusted[1]
Sweden	Reference	Reference	Reference	Reference	Reference	Reference
Central Europe, Eastern Europe and Central Asia	1.04 (0.97, 1.12)	0.96 (0.89, 1.03)	0.75 (0.71, 0.79)	0.73 (0.69, 0.78)	1.13 (1.03, 1.23)	1.11 (1.01, 1.21)
High income countries	0.88 (0.80, 0.98)	0.89 (0.80, 0.99)	0.88 (0.82, 0.94)	0.90 (0.84, 0.96)	1.10 (0.98, 1.23)	1.08 (0.97, 1.21)
Latin America and Caribbean	1.68 (1.43, 1.96)	1.52 (1.30, 1.76)	1.38 (1.22, 1.56)	1.33 (1.17, 1.51)	1.82 (1.50, 2.21)	1.70 (1.40, 2.07)
North Africa and Middle East	1.96 (1.87, 2.05)	1.52 (1.44, 1.60)	1.33 (1.27, 1.38)	1.14 (1.10, 1.20)	1.65 (1.55, 1.77)	1.53 (1.42, 1.64)
South Asia	1.34 (1.16, 1.56)	1.28 (1.10, 1.49)	1.21 (1.08, 1.36)	1.22 (1.09, 1.37)	1.54 (1.28, 1.85)	1.52 (1.27, 1.83)
Southeast Asia and East Asia	0.96 (0.85, 1.09)	0.80 (0.71, 0.91)	0.63 (0.57, 0.70)	0.62 (0.56, 0.69)	0.95 (0.81, 1.11)	0.89 (0.76, 1.04)
Sub-Saharan Africa	1.44 (1.33, 1.56)	0.92 (0.84, 0.99)	0.91 (0.85, 0.98)	0.71 (0.66, 0.77)	0.94 (0.83, 1.07)	0.80 (0.70, 0.91)

[1] Adjusted models included maternal age, parity, educational attainment and body mass index as covariates.

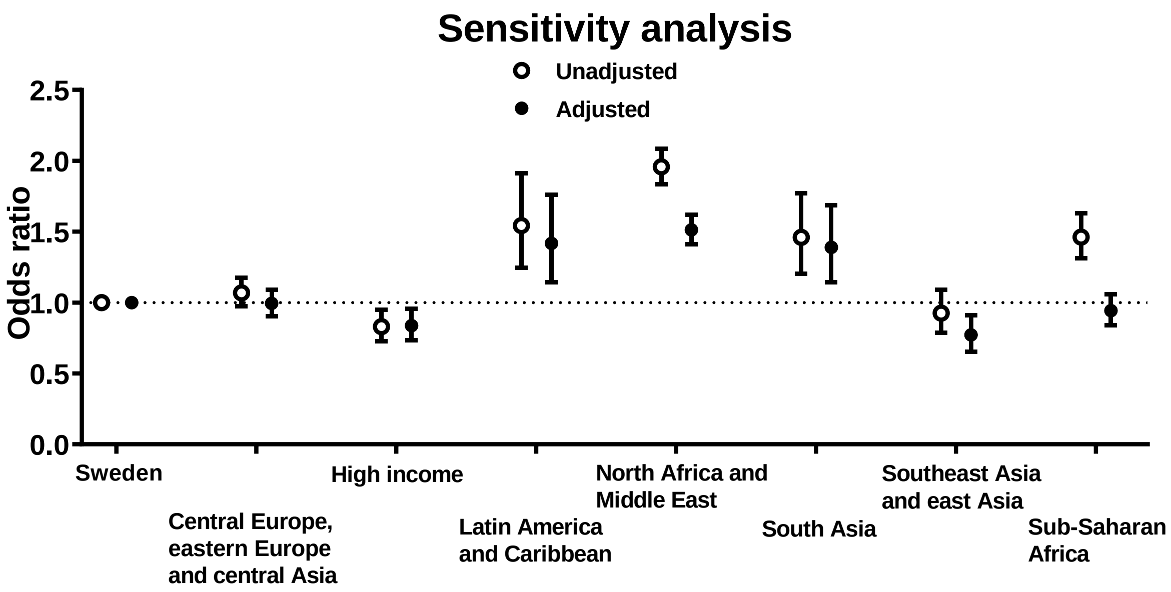

Figure S1. Sensitivity analysis showing the odds ratios of poor self-rated health before pregnancy by birth regions for women with **complete data of self-rated health at all time points, i.e. before, during and after pregnancy** (n = 398 025). Logistic regression was utilized to calculate odds ratios with 95% confidence intervals (women born in Sweden was the reference group). The adjusted model included maternal age, parity, educational attainment and body mass index as covariates.

Table S4. Odds ratios with 95 % confidences intervals for poor self-rated health before (n = 576 491), during (n = 432 266) and after pregnancy (n = 425 468) by birth regions with adjustments for maternal age, parity, educational attainment, body mass index as well as **occupation**.

	Poor self-rated health		
	**Before pregnancy**	**During pregnancy**	**After pregnancy**
	**Adjusted[1] + occupation[2]**	**Adjusted[1] + occupation[2]**	**Adjusted[1] + occupation[2]**
Sweden	Reference	Reference	Reference
Central Europe, Eastern Europe and Central Asia	0.93 (0.86, 1.00)	0.75 (0.70, 0.79)	1.07 (0.98, 1.18)
High income countries	0.86 (0.77, 0.95)	0.90 (0.84, 0.97)	1.05 (0.94, 1.18)
Latin America and Caribbean	1.50 (1.28, 1.77)	1.34 (1.18, 1.52)	1.64 (1.34, 1.99)
North Africa and Middle East	1.45 (1.37, 1.53)	1.19 (1.14, 1.25)	1.43 (1.33, 1.54)
South Asia	1.14 (0.98, 1.33)	1.25 (1.11, 1.40)	1.41 (1.17, 1.70)
Southeast Asia and East Asia	0.82 (0.72, 0.94)	0.64 (0.58, 0.71)	0.87 (0.74, 1.02)
Sub-Saharan Africa	1.02 (0.94, 1.11)	0.77 (0.72, 0.83)	0.78 (0.68, 0.89)

[1] Models included maternal age, parity, educational attainment and body mass index as covariates.

[2] Occupation was classified as i) employed ii) studying iii) parental leave iv) unemployed v) sick leave/disability pension vi) others

Table S5. Generalized linear mixed model with a time by region interaction for self-rated health before, during and after pregnancy[1].

	**Odds ratios**		**Ratio of odds ratios**			
	**Before pregnancy**		**During pregnancy**		**After pregnancy**	
	Unadjusted	Adjusted[1]	Unadjusted	Adjusted[1]	Unadjusted	Adjusted[1]
Sweden	Reference	Reference	Reference	Reference	Reference	Reference
Central Europe, Eastern Europe and Central Asia	1.05 (0.97, 1.13)	1.00 (0.93, 1.08)	0.71 (0.65, 0.78)	0.71 (0.65, 0.79)	1.07 (0.95, 1.21)	1.08 (0.96, 1.21)
High income countries	0.88 (0.79, 0.98)	0.88 (0.80, 0.98)	1.00 (0.88, 1.13)	1.00 (0.89, 1.14)	1.25 (1.07, 1.46)	1.26 (1.08, 1.47)
Latin America and Caribbean	1.67 (1.42, 1.96)	1.55 (1.32, 1.83)	0.82 (0.67, 1.01)	0.84 (0.68, 1.03)	1.08 (0.84, 1.39)	1.10 (0.85, 1.42)
North Africa and Middle East	1.94 (1.85, 2.04)	1.65 (1.57, 1.73)	0.68 (0.63, 0.72)	0.67 (0.63, 0.72)	0.83 (0.76, 0.90)	0.83 (0.76, 0.90)
South Asia	1.34 (1.14, 1.56)	1.32 (1.13, 1.54)	0.89 (0.74, 1.09)	0.90 (0.74, 1.09)	1.13 (0.88, 1.44)	1.13 (0.88, 1.44)
Southeast Asia and East Asia	0.98 (0.86, 1.11)	0.91 (0.80, 1.03)	0.65 (0.55, 0.76)	0.64 (0.55, 0.76)	0.99 (0.81, 1.21)	0.99 (0.80, 1.21)
Sub-Saharan Africa	1.45 (1.34, 1.57)	1.08 (0.99, 1.17)	0.63 (0.56, 0.70)	0.63 (0.57, 0.70)	0.65 (0.56, 0.75)	0.65 (0.56, 0.76)

[1]An additional analysis of the primary outcome was conducted using generalized linear mixed model with a time by region interaction. Sweden was used as the reference region, and before pregnancy was considered baseline. Random effects were added for the intercept and for the time covariates. An unadjusted and an adjusted model was fitted (with the same adjustments as in the primary analysis). The ratio of odds ratio estimates in the table provide the value by how much the before odds ratio should be multiplied with to receive the during/after odds ratio. For instance, in the unadjusted model, the odds ratio before pregnancy comparing North Africa and Middle East with Sweden was 1.94, and so to receive the odds ratio between these two regions during pregnancy we multiply with 0.68, i.e. 1.94 * 0.68 = 1.32. In this way, we can view the ratio of odds ratios as the magnitude of change. Looking at the tables we can see that some of these magnitudes are statistically significant (does not contain 1 in the confidence interval), while we cannot rule out the null value in some cases.

[2] Adjusted models included maternal age, parity, educational attainment and body mass index as covariates.
